# Multi-Mode Binding of Cellobiohydrolase Cel7A from *Trichoderma reesei* to Cellulose

**DOI:** 10.1371/journal.pone.0108181

**Published:** 2014-09-29

**Authors:** Jürgen Jalak, Priit Väljamäe

**Affiliations:** Institute of Molecular and Cell Biology, University of Tartu, Tartu, Estonia; Russian Academy of Sciences, Institute for Biological Instrumentation, Russian Federation

## Abstract

Enzymatic hydrolysis of recalcitrant polysaccharides like cellulose takes place on the solid-liquid interface. Therefore the adsorption of enzymes to the solid surface is a pre-requisite for catalysis. Here we used enzymatic activity measurements with fluorescent model-substrate 4-methyl-umbelliferyl-β-D-lactoside for sensitive monitoring of the binding of cellobiohydrolase *Tr*Cel7A from *Trichoderma reesei* to bacterial cellulose (BC). The binding at low nanomolar free *Tr*Cel7A concentrations was exclusively active site mediated and was consistent with Langmuir's one binding site model with *K*_d_ and *A*_max_ values of 2.9 nM and 126 nmol/g BC, respectively. This is the strongest binding observed with non-complexed cellulases and apparently represents the productive binding of *Tr*Cel7A to cellulose chain ends on the hydrophobic face of BC microfibril. With increasing free *Tr*Cel7A concentrations the isotherm gradually deviated from the Langmuir's one binding site model. This was caused by the increasing contribution of lower affinity binding modes that included both active site mediated binding and non-productive binding with active site free from cellulose chain. The binding of *Tr*Cel7A to BC was found to be only partially reversible. Furthermore, the isotherm was dependent on the concentration of BC with more efficient binding observed at lower BC concentrations. The phenomenon can be ascribed to the BC concentration dependent aggregation of BC microfibrils with concomitant reduction of specific surface area.

## Introduction

Cellulose is the most abundant polysaccharide on Earth and is an appealing raw material for many biotechnological applications. As a structural polysaccharide, cellulose has evolved to heterogeneous structure that makes it recalcitrant towards chemical as well as enzymatic degradation [1]. Individual cellulose chains adhere with each other by hydrogen bonding and van der Waals interactions forming crystalline microfibrils. Enzymatic depolymerization of cellulose chains is carried out by cellulases and it takes place in the solid-liquid interface. Thus the adsorption of cellulases to cellulose surface is a prerequisite for catalysis [2], [3]. To facilitate interactions with substrate many cellulases have modular structure consisting of a catalytic domain (CD) that is connected through a linker peptide with a smaller carbohydrate binding module (CBM) [4]. The best studied cellulase is a processive cellobiohydrolase *Tr*Cel7A from *Trichoderma reesei* (*Hypocrea jecorina*). The tunnel shaped active site of *Tr*Cel7A resides in CD and accommodates 10 consecutive glucose units along cellulose chain. It has been shown that separated CBM and CD of *Tr*Cel7A can both bind to cellulose [5]. Furthermore, the linker peptide also contributes to binding [6], [7]. The binding of cellulases is also influenced by the properties of cellulose, which is heterogeneous at different levels: content of different crystalline allomorphs, different crystal faces, amorphous regions, degree of polymerization, content and size of pores, specific surface area etc [3]. In the case of lignocellulosic substrates the binding is further complicated by the presence of lignin and hemicelluloses [8]. Therefore pure model celluloses like bacterial cellulose (BC) and bacterial microcrystalline cellulose (BMCC) are often used in binding studies of cellulases. Modular architecture of the enzymes in combination with heterogeneous substrate is expected to result in a complex binding isotherm. Despite this expected complexity the Langmuiŕs one binding site model has been often found to be sufficient to describe cellulase binding [3], [9]. Among alternative models the Langmuiŕs two independent binding site model [10], [11]. Freundlich model [12], [13], and combined Langmuiŕs-Freundlich model or Hilĺs cooperative binding model [14] have also been used. Although direct measurement of the bound enzyme has been reported [15] the most often applied experimental approach is the depletion method, where the concentration of free enzyme is measured and the amount of cellulose bound enzyme is found as a difference between the concentration of total and free enzyme. The simplest method for the measurement of the concentration of free enzyme is based on the absorbance or the fluorescence measurement of the enzyme protein. However, measurements of cellulase binding are complicated by the possible non-specific adsorption of enzymes to reaction vessels [16]. This may lead to the overestimation of the binding strength, especially at low cellulase concentrations. Therefore blocking agents like bovine serum albumin (BSA) are often used to circumvent the problem. However, in the presence of BSA, the methods relying on direct quantification of protein are not applicable for the measurement of cellulose free cellulase. To measure the binding in the presence of BSA radioactivity [17], [18] or fluorescence labeled cellulases [19] or chimeras of CBMs with fluorescent proteins [14], [20] have been used. Recently we have developed a sensitive method relying on the activity measurement of *Tr*Cel7A with low Mw model substrates to monitor the binding of *Tr*Cel7A [21], [22]. Since activity measurements reveal the concentration of enzyme with free active site the method can be used to distinguish between different populations of bound enzyme: total bound enzyme, bound through the active site, and bound but with free active site [21], [23]. Here we used fluorescent model substrate, 4-methyl-umbelliferyl-β-D-lactoside (MUL), to study the binding of *Tr*Cel7A to BC. Sensitive detection enabled to measure sub-nanomolar concentrations of *Tr*Cel7A with free active sites and to reveal the strongest binding observed with non-complexed cellulases.

## Results and Discussion

### Measuring the binding of *Tr*Cel7A to BC

Binding of cellulases to the cellulose surface is a prerequisite step before catalysis and has been in the focus of numerous studies. Despite intensive research there is a gap in our knowledge in many issues like contribution of different binding modes in binding of modular enzymes. This has lead to different and often controversial hypotheses about the role of binding in controlling the overall rate of cellulose degradation [21]–[28]. Beside the nature of enzyme and substrate, the method used for binding measurements along with the range of enzyme and substrate concentrations included in the measurements seems to be important [29]. Binding measurements imply the separation of cellulose bound and free cellulase. Here, the suitability of two methods for this purpose, centrifugation and filtration, were tested. Filtration was found to be more accurate as there was an initial rapid release of *Tr*Cel7A from cellulose pellet after centrifugation resulting in the overestimation of the concentration of *Tr*Cel7A free from cellulose ([*Tr*Cel7A]_Free_) (Figure S1 in File S1). In this study we used the enzymatic activity of *Tr*Cel7A with MUL substrate to measure [*Tr*Cel7A]_Free_. Hydrolysis of MUL by *Tr*Cel7A results in the formation of lactose and 4-methylumbelliferone (MU). The latter can easily be detected by the fluorescence at high pH. Advantages of this approach are high sensitivity and applicability in the presence of BSA used to prevent non-specific binding. Control experiments judged that in the presence of BSA (0.1 g/L) there was no non-specific binding of *Tr*Cel7A to the reaction vessels and filters. BSA was also found to be necessary to avoid the binding of MU to BC. A certain disadvantage of using activity measurements with MUL is the necessity to remove cellobiose released from cellulose hydrolysis to avoid the cellobiose inhibition of MUL hydrolysis by *Tr*Cel7A [21], [22]. Therefore, before the analysis of MUL activity, the filtrates containing *Tr*Cel7A_Free_ were treated with β-glucosidase, which hydrolyzes cellobiose into two molecules of glucose. Glucose inhibition of *Tr*Cel7A is weak [30] and can be neglected under our experiment conditions. Beside sensitive detection of low enzyme concentrations a virtue of using activity with MUL is the possibility to discriminate between the populations of total bound *Tr*Cel7A (*Tr*Cel7A_Bound_) and *Tr*Cel7A bound through the active site (*Tr*Cel7A_Bound-OA_). The latter can be measured by following the inhibition of MUL hydrolysis by cellulose [22], [31]. The subscript OA refers to occupied active site i.e. the active site is occupied by cellulose chain and not available for MUL hydrolysis. Accordingly the subscript FA refers to free active site for MUL hydrolysis. The population of bound *Tr*Cel7A with free active sites (*Tr*Cel7A_Bound-FA_) is found from the difference between total bound *Tr*Cel7A and *Tr*Cel7A_Bound-OA_, [*Tr*Cel7A]_Bound-FA_ = [*Tr*Cel7A]_Bound_ - [*Tr*Cel7A]_Bound-OA_. Beside MUL, other low-Mw model substrates like para-nitrophenyl-β-D-lactoside (pNPL) [21]–[23] have been used to measure the active site mediated binding of *Tr*Cel7A.

The measurement of binding kinetics to BC revealed no changes in [*Tr*Cel7A]_Free_ within studied incubation times (0.5 – 5 h) (Figure S2 in File S1) and equilibration times between 10 min and 30 min were used in further binding experiments. Although literature reports support fast binding of cellulases to BC and BMCC with equilibrium times in the range of few minutes [10], [25] we used somewhat longer incubation times to reveal possible slow irreversible binding. Constant levels of bound *Tr*Cel7A observed over long incubation period (Figure S2 in File S1) indicates that the enzymatic activity of *Tr*Cel7A does not interfere with binding. This is in accord with the processive mode of hydrolysis whereby the cellulose crystal is degraded at its surface, “layer by layer”, resulting in thinner crystals with no significant changes in total surface area [11], [32], [33].

### Binding of *Tr*Cel7A to BC involves multiple binding modes with different affinities

**Figure 1:**
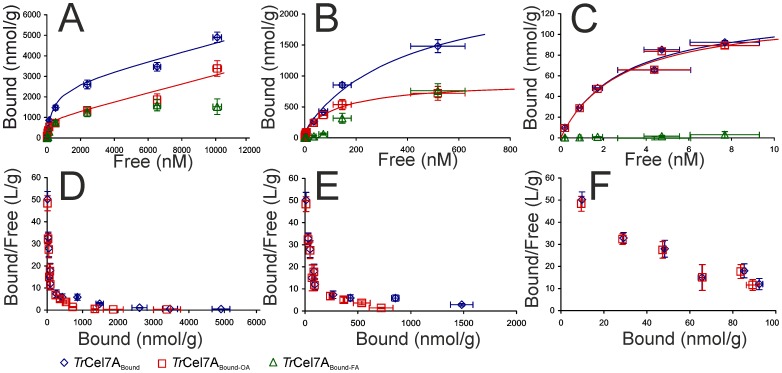
Variation of free enzyme concentration over 4 orders of magnitude reveals multi-mode binding of *Tr*Cel7A to BC. [Free] – [Bound] plots (A – C) and Scatchard plots (D – F) of binding of *Tr*Cel7A to BC (1 g/L). Full binding isotherm is dissected into three regions with different dominating binding modes. The low affinity binding mode dominates at free *Tr*Cel7A concentrations ([*Tr*Cel7A]_Free_) above 1.0 µM (A and D). The medium affinity binding mode dominates in the 0.1 µM – 1.0 µM range of [*Tr*Cel7A]_Free_ (B and E). The high affinity binding mode dominates at [*Tr*Cel7A]_Free_ up to 10 nM (C and F). Total bound *Tr*Cel7A (*Tr*Cel7A_Bound_), *Tr*Cel7A bound through the active site (*Tr*Cel7A_Bound-OA_), and bound *Tr*Cel7A with free active site (*Tr*Cel7A_Bound-FA_). Solid lines represent best fits of Langmuiŕs one (C), two (B), and three (A) independent binding site model. Error bars are at least from three independent measurements.

Binding measurements made by varying the concentration of *Tr*Cel7A over 4 orders of magnitude (BC was at 1 g/L) revealed complex binding (Figure 1). None of the equations tested provided a very good fit if the full dataset (n = 82) was used in the analysis. Equations tested included Langmuiŕs one (R = 0.9764), two (R = 0.9932), and three independent binding site models (R = 0.9932), Freundlich model (R = 0.9896), Hilĺs cooperative binding model (R = 0.9896), and sum of Langmuiŕs and Hilĺs model (R = 0.9942). Although some models provided a reasonably good fit with R values above 0.99, there was a strong systematic deviation between the experiment and the model in the low-nanomolar range of free *Tr*Cel7A concentrations (Figure S3 in File S1). Restricting the dataset with the highest [*Tr*Cel7A]_Free_ up to 10 nM resulted in a good accord with Langmuiŕs one binding site model (Figure 1C and F) with *K*_d_ = 2.9 nM and *A*_max_ = 126 nmol/g. Next we analyzed the binding in the range of [*Tr*Cel7A]_Free_ up to 1.0 µM using the Langmuiŕs two binding sites model (Figure 1B and E). In analysis, the parameters values for the first, high affinity binding mode were fixed (*K*_d_ = 2.9 nM and *A*_max_ = 126 nmol/g) and the parameters values for the second binding mode were found by fitting. The *K*_d_ for the second, medium affinity binding mode was more than two orders of magnitude higher than that for the high affinity binding mode (Table 1). Finally, the full dataset was analyzed using the Langmuiŕs three binding sites model (Figure 1A and D). Here, the parameters values for the first two binding modes were fixed. Because the third, low affinity binding mode is far from saturation, only the *A*_max_/*K*_d_ value can be found for this binding mode (Table 1). It must be noted that although the differences in R values between two and three binding site model does not justify the use of more complex model, the visual inspection of the fits suggests that the presence of at least three binding sites must be assumed to describe the data (Figure S3 in File S1).

**Table 1:** Binding isotherm parameters for different binding modes of active site mediated and total binding of cellobiohydrolase *Tr*Cel7A to bacterial cellulose.

	High affinity binding mode^b^		Medium affinity binding mode^c^		Low affinity binding mode^d^	
Parameter^a^	Total bound	Active site bound	Total bound	Active site bound	Total bound	Active site bound
*A*_max_ (nmol/g)	126±17	122±14	2440±260	790±50	-	-
*K*_d_ (nM)	2.9±1.0	2.8±0.8	406±82	156±25	-	-
*A*_max_/*K*_d_ (L/g)	43.1±14.7	43.3±12.7	6.0±1.2	5.1±0.8	0.21±0.01	0.22±0.01

a Parameter values were found by non-linear regression analysis of data in Figure 1. Error limits are the parameter errors from the non-linear regression route and are not primarily statistical in origin.

b Dataset was restricted with [*Tr*Cel7A]_Free_ up to 10 nM (Figure 1C) and Langmuiŕs one binding site model was used in analysis.

c Dataset was restricted with [*Tr*Cel7A]_Free_ up to 1.0 µM (Figure 1B) and Langmuiŕs two binding site model was used in analysis. Parameter values for the first, high affinity binding mode were fixed and the parameter values for the second, medium affinity binding mode were found by non-linear regression analysis.

d Full dataset (n = 82) was analyzed according Langmuiŕs three binding site model (Figure 1A). Parameter values for the first two binding modes were fixed and the parameter values for the third, low affinity binding mode were found by non-linear regression analysis. Because of the low degree of saturation of this binding mode only the value of *A*_max_/*K*_d_ can be found.

The active site mediated binding of *Tr*Cel7A to BC was measured in parallel with the total bound *Tr*Cel7A to reveal the contribution of non-productive binding modes, where the enzyme is attached to BC without cellulose chain in the active site. In the range of low-nanomolar [*Tr*Cel7A]_Free_ the enzyme was bound exclusively through the active site (Figure 1C and F). However, in the range of [*Tr*Cel7A]_Free_ between 0.1 – 1.0 µM the contribution of bound enzyme with free active site (*Tr*Cel7A_Bound-FA_) in total bound enzyme was significant (Figure 1B and E). Somewhat surprisingly we found that the low affinity binding that dominates in the high micromolar range of [*Tr*Cel7A]_Free_ was active site mediated (Figure 1A and D). Similarly to the total binding none of the tested equations were sufficient to describe the active site mediated binding if the full dataset was used in the analysis. The parameter values for the active site mediated binding were found analogously to those for the total bound enzyme and are listed in Table 1. The presence of different populations of bound enzyme further supports the necessity to include at least three binding modes in the analysis of experiment data. We have two populations of active site bound enzyme with approximately 200 fold different *A*_max_/*K*_d_ values (Table 1) and we also have the population of bound enzyme with free active site that is qualitatively different (Figure 1A and B). Earlier attempts to measure active site mediated binding have shown that the majority of *Tr*Cel7A is bound through the active site and the population of *Tr*Cel7A_Bound-FA_ constitutes usually less than 10% of the total bound enzyme [21], [23]. In contrast, bound enzyme with the free active site has been proposed to be the dominating binding mode of Cel9A from *Thermobifida fusca* [34]. The data from this study demonstrate that the contribution of active site mediated binding depends primarily on the enzyme to substrate ratio.

**Figure 2:**
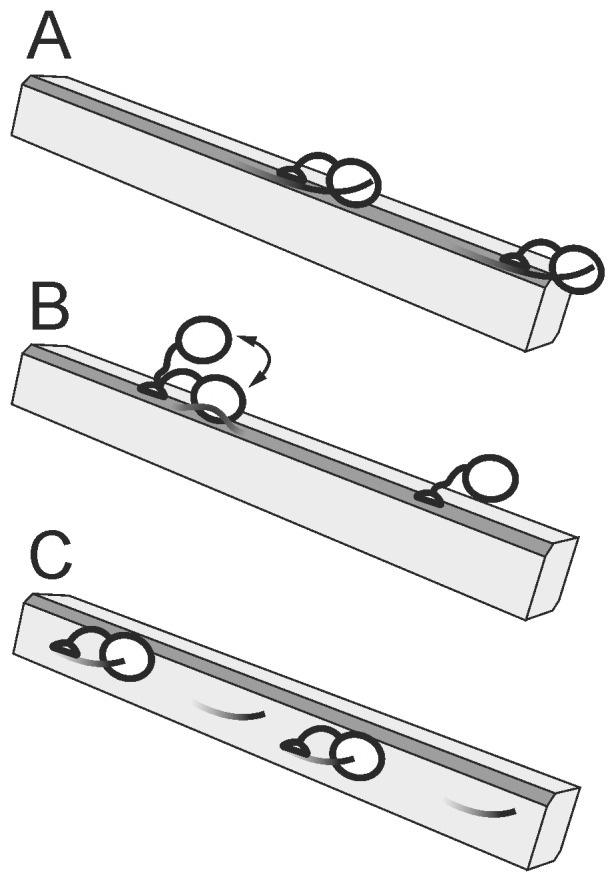
Proposed productive and non-productive interactions between *Tr*Cel7A and BC. (A) The high affinity binding mode corresponds to the productive binding to cellulose chain ends on the hydrophobic face of BC microcrofibril through both domains, CBM and CD. (B) The medium affinity binding mode includes non-productive binding to the hydrophobic face, where enzyme is attached through CBM only. Latter can become productive binding upon disengagement of cellulose chain into the active site by endo-mode attack. (C) The low affinity binding mode may correspond to the active site mediated binding to cellulose chain ends on the hydrophilic face. The hydrophobic face of cellulose microfibril is shown in dark gray and the cellulose chain ends available for binding through CD are depicted as protruding lines. The *A*_max_ and *K*_d_ values for corresponding binding modes are listed in Table 1.

Figure 2 shows the proposed mechanistic interpretation of binding modes with different affinity. It has been demonstrated by experiment [35] and also by molecular dynamics simulation [36] that CBM of *Tr*Cel7A preferentially binds to the hydrophobic face of cellulose crystal. *Tr*Cel7A degrades cellulose crystal also from its hydrophobic face [33]. The estimated surface area of the hydrophobic face is approximately 100 µmoles of cellobiose units per gram BC [10]. Considering the average degree of polymerization of our BC preparation of 825±10 cellobiose units, one can estimate that there is 0.12 µmoles of reducing ends/g BC on the hydrophobic face. This is in good accord with the *A*_max_ values of the high affinity binding mode for both the total bound and the active site bound *Tr*Cel7A (Table 1). Since in this region of the isotherm all of the bound enzyme was bound through the active site (Figure 1C and F) we propose that the high affinity binding mode represents the productive binding to the chain ends on the hydrophobic face, i.e. *Tr*Cel7A is attached through both domains, CBM and CD, and the cellulose chain is engaged into the active site tunnel (Figure 2A).

The medium affinity binding dominates in the range of free *Tr*Cel7A concentrations between 0.1 µM and 1.0 µM. In this range both populations, the active site bound *Tr*Cel7A and the bound *Tr*Cel7A with the free active site, were present (Figure 1B and E). CBM of *Tr*Cel7A covers 10 cellobiose units [37], whereas CD covers approximately 48 cellobiose units [38]. Thus, for CBM, the binding capacity of the hydrophobic face is expected to be 10 µmoles CBM per gram BC (100 µmoles of cellobiose units per gram BC/10 cellobiose units per CBM). The corresponding figure for CD is approximately 2 µmoles per gram BC. Thus, the binding capacity 2.4 µmol/g found for the total bound enzyme in the medium affinity binding mode (Table 1) is in the same order with the estimated binding capacity of *Tr*Cel7A on the hydrophobic face. We propose that in the range of medium affinity binding there is a balance between two populations of bound enzyme. The population of bound *Tr*Cel7A with the free active site apparently represents the non-productive binding to the hydrophobic face, where the enzyme is attached through CBM only (Figure 2B). Because *Tr*Cel7A can employ an endo-mode initiation on BC [39] the initially non-productive binding through CBM can become productive binding after disengagement of cellulose chain from the crystal lattice to the active site (Figure 2B). The medium affinity active site mediated binding (Table 1) may thus represent the endo-mode complexation of *Tr*Cel7A with cellulose chains on the hydrophobic face.

The third, low affinity binding mode dominates in the free *Tr*Cel7A concentrations above 1.0 µM (Figure 1A and D). Since its binding capacity is high, it may correspond to the binding of *Tr*Cel7A to the hydrophilic face of cellulose crystal (Figure 2C). Because of the large specific area of the hydrophilic face and low affinity we were not able to saturate the low affinity binding mode in our experiments. A recent molecular dynamics study demonstrated that *Tr*Cel7A CBM can bind also to the hydrophilic face, although it will diffuse to the hydrophobic face upon vacancy [36]. However, as evidenced by the very similar *A*_max_/*K*_d_ values for the total and the active site mediated binding (Table 1), the low affinity binding mode is active site mediated. One can speculate that the low affinity binding represents the active site mediated binding to the chain ends on the hydrophilic face. Because cellulose chains in the hydrophilic face are more tightly associated with the crystal lattice their disengagement to the enzymes active site is energetically less favorable resulting in a high *K*_d_ value.

It must be noted that the above mechanistic interpretation was only a simplified view to the binding of *Tr*Cel7A to BC. Earlier studies have shown that the analysis of cellulase binding is complicated by the presence of the overlapping binding sites [38] and cooperative effects [14]. This additional complexity may reveal in the insufficiency of Langmuiŕs three independent binding sites model in describing the full dataset. Although the analysis of our binding data in the region of high and medium affinity binding mode using Hill’s plot suggested no cooperative effects within these binding modes (Figure S4 in File S1) more detailed studies are needed to reveal the possible interdependency between different binding modes. The *K*_d_ value of 2.9 nM measured here for the high affinity binding mode (Figure 1C and F, Figure 2A) is among the strongest bindings observed with non-complexed cellulases. Using radioactivity labeled proteins the *A*_max_/*K*_d_ values of 18 L/g have been reported for the binding of *Tr*Cel7A and *Tr*Cel6A to BMCC at 22 °C [18]. Using fluorescence measurements of low free enzyme concentrations, Herner et al. [40] reported the *K*_d_ value of 33±6 nM (at 20°C) for binding of *Tr*Cel7A to microcrystalline cellulose. The binding of CBM of exoglucanase Cex from *Cellulomonas fimi* to BMCC with *K*_d_ value of 16±3.6 nM (at 30°C) has been measured using isothermal titration calorimetry [41]. Using the measurements of fluorescence recovery after photobleaching Moran-Mirabal et al. [42] have reported *K*_d_ values in the range of 10 – 50 nM for binding of *Thermobifida fusca* cellulases to BMCC. However, most of the reported *A*_max_ and *K*_d_ values for the binding of different cellulases to BC or BMCC are in the same order with corresponding values found here for the medium affinity binding mode (Figure 1B and E, Figure 2B, Table 1) [7], [10], [43]. The *K*_d_ values reported for the binding of *Tr*Cel7A to other crystalline celluloses like Avicel, algal celluloses, and cellulose III are also in the sub- or low-micromolar range [5], [11], [13], [14], [26], [44]–[49]. The sensitivity of the methods like protein absorbance apparently sets the limits for the lowest observable free enzyme concentration and the possible low capacity strong binding modes may remain undetectable. The results presented here support the conclusions of a recent single molecule tracking study that the performance of *Tr*Cel7A measured at low nanomolar enzyme concentrations is strikingly different from that measured at micromolar concentrations [29]. The conclusion from above is that measurements at very low enzyme concentrations have to be included in studies aiming to measure the rate constants for different steps of the hydrolysis of the insoluble substrate like association, processive movement and dissociation.

### Reversibility of the binding of *Tr*Cel7A to BC

**Figure 3:**
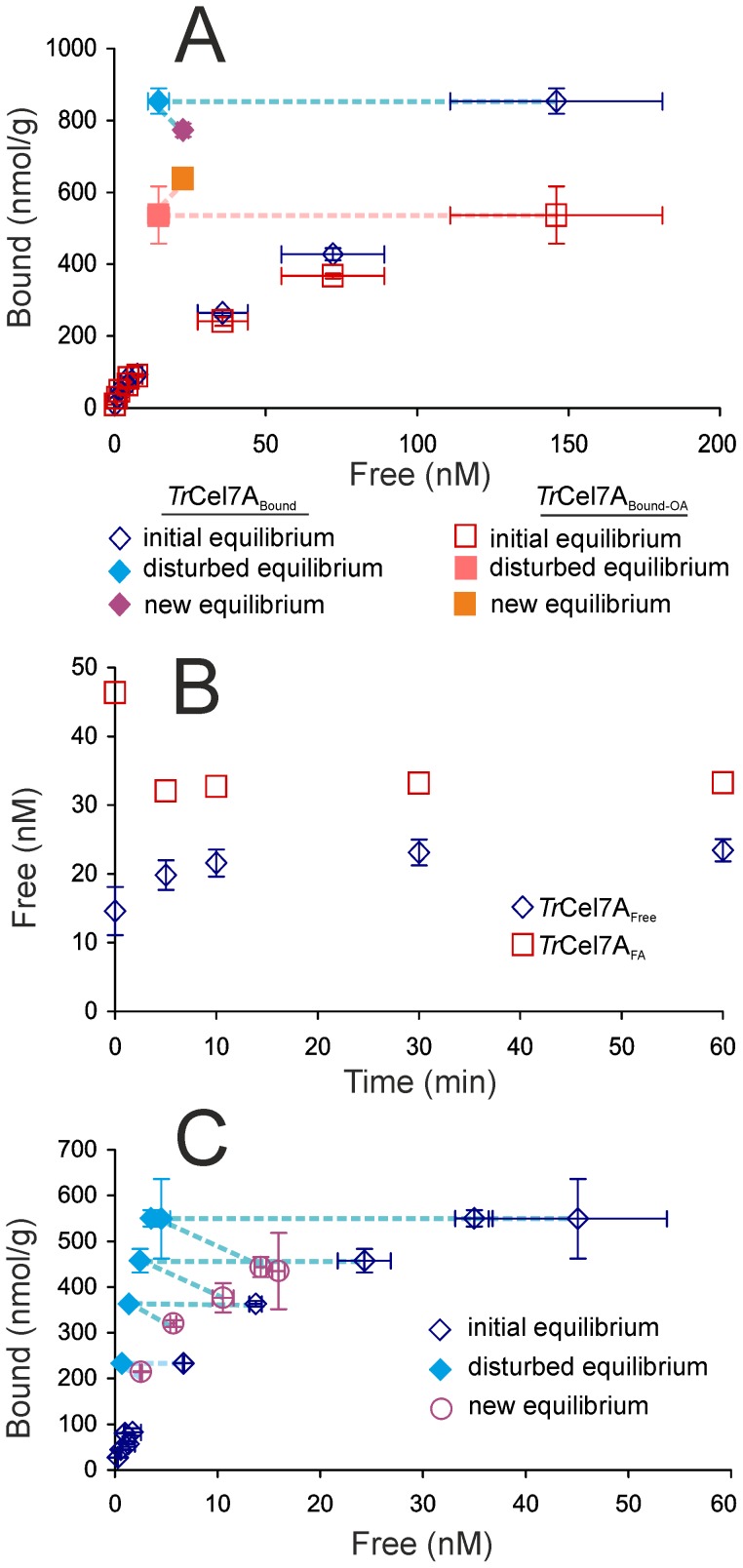
Binding of *Tr*Cel7A to BC is only partially reversible. (A and B) Dilution experiment. BC at 1.0 g/L was incubated with 1.0 µM *Tr*Cel7A to establish equilibrium. Equilibrium was disturbed by the addition of buffer to bring up tenfold dilution and relaxation to new equilibrium was followed. (A) Binding reversibility was assessed in the basis of both, total bound *Tr*Cel7A, *Tr*Cel7A_Bound_ and active site bound *Tr*Cel7A, *Tr*Cel7A_Bound-OA_. Dotted lines show the progression from the initial equilibrium to the disturbed equilibrium to the new equilibrium. (B) Change in the concentration of *Tr*Cel7A free from cellulose, *Tr*Cel7A_Free_ and *Tr*Cel7A with free active site *Tr*Cel7A_FA_ in time after disturbance of equilibrium by dilution. (C) Free enzyme depletion experiment. BC at 0.1 g/L was incubated with *Tr*Cel7A at different concentrations to establish equilibrium. Cellulose with bound enzyme was pelleted by centrifugation, 90% of the supernatant was withdrawn and pellet was resuspended in the same amount of buffer to disturb the equilibrium. The position of the new equilibrium was measured after 30 min from the disturbance. Binding reversibility was assessed in the basis of total bound *Tr*Cel7A. Dotted lines show the progression from the initial equilibrium to the disturbed equilibrium to the new equilibrium.

Binding reversibility of cellulases has been a focus of numerous studies but the results have been often controversial. In some studies full reversibility is observed [17], [18], [43] whereas others report significant hysteresis [18], [42], [43], [50]–[54]. Here we studied the reversibility of the binding of *Tr*Cel7A using two different approaches, dilution and supernatant replacement experiment. In the dilution experiment BC at 1 g/L was equilibrated with 1.0 µM *Tr*Cel7A for 30 min, after which the equilibrium was disturbed by the addition of buffer to bring a tenfold increase in the total volume. Using parallel experiments the binding reversibility was assessed in the basis of both *Tr*Cel7A_Bound_ and *Tr*Cel7A_Bound-OA_ (Figure 3A).

The change in the concentration of *Tr*Cel7A_Free_ and *Tr*Cel7A_FA_ in time after the disturbance of equilibrium by dilution is shown in Figure 3B. In the case of *Tr*Cel7A_Free_ there was a rapid increase in its concentration during first 5 min after the dilution with no further significant changes indicating the relaxation to the new equilibrium. In contrast to *Tr*Cel7A_Free_, there was a decrease in the concentration of *Tr*Cel7A_FA_ after dilution resulting in a new equilibrium with lower [*Tr*Cel7A_FA_] (Figure 3B). This indicates an increase in the contribution of the active site mediated binding to the total bound enzyme in the new equilibrium, which is in qualitative agreement with the increased contribution of *Tr*Cel7A_Bound-OA_ in the region of low [*Tr*Cel7A]_Free_ (Figure 1). In the case of reversible binding the new equilibrium position established after dilution is expected to fall into the initial isotherm. However, the position of the new equilibrium remained far from the initial isotherm, indicating strong hysteresis. In the case of *Tr*Cel7A_Bound-OA_ the amount of bound enzyme even increased upon dilution (Figure 3A). We also assessed the binding reversibility (at the level of total bound *Tr*Cel7A) by disturbing the equilibrium without changing the concentration of BC. For that, BC at 0.1 g/L was equilibrated with *Tr*Cel7A at different concentrations for 30 min. After equilibration cellulose was pelleted by centrifugation and 90% of the *Tr*Cel7A_Free_ containing supernatant was replaced with fresh buffer to disturb the equilibrium. The position of new equilibrium was measured 30 min after the disturbance. The new isotherm did not overlap with the initial isotherm but the hysteresis was less prominent than in the case of the dilution experiment (Figure 3C). The difference between two approaches was that in the case of dilution experiment cellulose was diluted in parallel with *Tr*Cel7A_Free_, whereas in the supernatant exchange experiment cellulose concentration was not changed. This prompted us to measure the binding isotherms at different BC concentrations.

### Binding isotherm of *Tr*Cel7A depends on the concentration of BC

**Figure 4:**
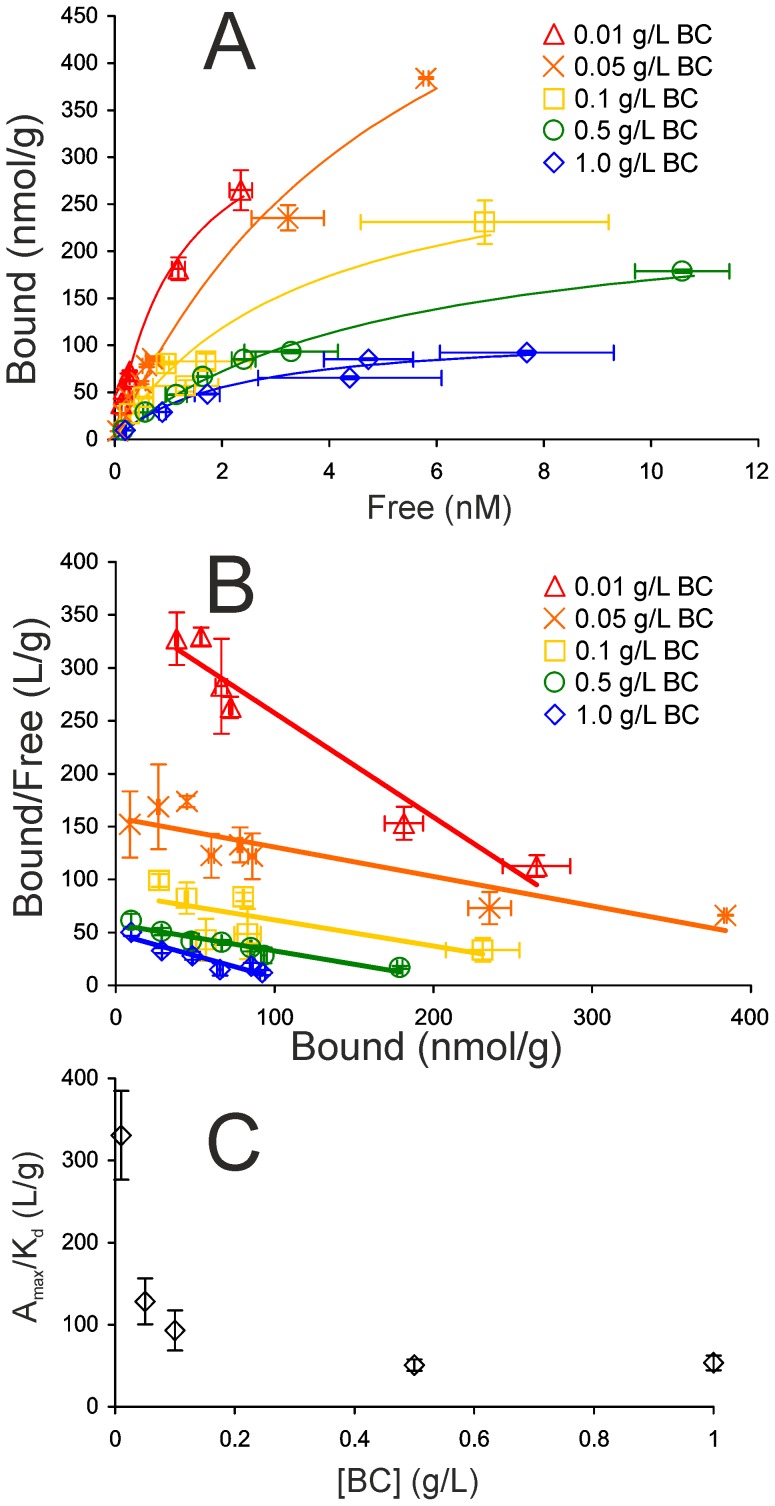
Binding isotherm of *Tr*Cel7A depends on BC concentration. Binding isotherms (A), and corresponding Scatchard plots (B) of binding of *Tr*Cel7A (in the level of total bound *Tr*Cel7A) to BC at different concentrations. Solid lines represent best fits of Langmuiŕs one binding site model. Error bars are at least from three independent measurements. (C) *A*_max_/*K*_d_ values at different BC concentrations.

Binding isotherms of *Tr*Cel7A (on the total bound enzyme basis) were measured at 5 different BC concentrations between 0.01 – 1.0 g/L. Here we focused only on the high binding affinity region of the isotherm ([*Tr*Cel7A]_Free_ up to 10 nM), where the binding was reasonably well consistent with the Langmuiŕs one binding site model (Figure 4A and B). As seen in Figure 4 the isotherms were dependent on the concentration of BC with more efficient binding observed at lower BC concentrations. Plotting the values of *A*_max_/*K*_d_ as a function of BC concentration revealed inverse relationship (Figure 4C). The dependence of *A*_max_ and *K*_d_ on BC concentration was less evident than that of *A*_max_/*K*_d_ (Figure S5 in File S1). It must be noted however, that because of the complex binding the determination of the *A*_max_ and *K*_d_ values is error prone. Although the Langmuiŕs one binding site model was sufficient to describe the binding at low nanomolar [*Tr*Cel7A]_Free_, further increase in [*Tr*Cel7A]_Free_ leads to an increased contribution of the medium affinity binding mode and systematic deviation from the one binding site model (Figure 1). For this reason *A*_max_ and *K*_d_ values depend on the highest concentration of *Tr*Cel7A_Free_ included into the analysis, which is always somewhat arbitrary. However, *A*_max_/*K*_d_ is given by the initial slope of the isotherm and is less sensitive to the highest concentration of *Tr*Cel7A_Free_ included into the analysis.

The binding isotherm of cellulases is expected to be independent of the concentration of cellulose. This expectation relies on the assumption, that the total amount of binding sites per gram cellulose as well as binding affinity are constants independent of cellulose concentration. Although the binding isotherms of cellulases have been in the focus of numerous studies, the isotherms are almost exclusively measured at one cellulose concentration. However, Wang et al. studied the binding of crude cellulase to Avicel and found also, that the binding was stronger at lower Avicel concentration [55]. A possible mechanistic interpretation would be that there is a cellulose concentration dependent association of cellulose microfibrils with concomitant decrease in specific surface area available for binding. In recent study Cruys-Bagger et al. proposed that cellulose concentration dependent reduction in the surface area was responsible for lower association rate constants between cellulose and *Tr*Cel7A observed at higher cellulose concentrations [28]. Consistent with the binding data reported here a recent scanning electron microscopy study by Kuijk et al. demonstrated the BC concentration dependent formation of large flocks and aggregates in BC suspensions [56]. Association of BC microfibrils should lead to the exclusion of some of the surface area for the binding of cellulases. According to this scenario it is the *A*_max_ that should decrease with increasing cellulose concentration, whereas *K*_d_ is expected to remain unaffected. Although the mechanism of the reduced binding efficiency with increasing cellulose concentration remains to be studied, the underlying property of cellulose suspension is expected to depend on cellulose concentration according to that of the *A*_max_/*K*_d_ in Figure 4C. In regard to lignocellulose hydrolysis the data presented in Figure 4 support the suggestion that complete removal of hemicelluloses and lignin during biomass pretreatment may cause the association of cellulose microfibrils with concomitant reduction in surface area available for the binding of cellulases and may thus become a disadvantage [57]. Cellulose concentration dependent association of microfibrils may also contribute in the so called “solids effect” – a decrease in the degree of cellulose conversion with increasing cellulose concentration at constant cellulase to cellulose ratio [58]–[60].

## Materials and Methods

### Materials

MUL, pNPL and BSA were from Sigma-Aldrich and were used as purchased. *Tr*Cel7A was purified from the culture filtrate of *Trichoderma reesei* QM 9414 as described before [61]. *Aspergillus* β-glucosidase was purified from Novozyme 188 (Sigma C6105) according to [62]. Enzyme concentration was determined from absorbance at 280 nm using extinction coefficients of 84,400 M^−1^ cm^−1^ for *Tr*Cel7A and 180,000 M^−1^ cm^−1^ for β-glucosidase. BC was prepared by laboratory fermentation of *Gluconobacter xylinum* strain ATCC 53582 as described before [63]. Degree of polymerization of BC was 1650±20 glucose units as judged by the amount of reducing groups on BC measured using modified bicinchoninic acid method [63], [64].

### General conditions

All binding experiments were made in 50 mM sodium acetate buffer pH 5.0 (supplemented with 0.1 g/L BSA) at 25°C. If not stated otherwise the experiments were performed in 1.5 mL polypropylene microcentrifuge tubes without stirring.

### Measurement of the concentration of cellulose free *Tr*Cel7A

After separation of cellulose bound *Tr*Cel7A the concentration of cellulose free *Tr*Cel7A was measured by its MUL hydrolyzing activity. Before the activity measurement with MUL 0.4 mL samples were supplemented with β-glucosidase (final concentration 4 nM) and incubated overnight to remove any cellobiose released from the hydrolysis of BC. For MUL-ase activity measurements 0.41 mL of suitably diluted β-glucosidase treated sample was added to 5 µL of 0.5 mM MUL and incubated at 35°C for 0.5 h – 6 h depending on the concentration of *Tr*Cel7A. Dilution factors and incubation times were adjusted so that the rate of MUL hydrolysis corresponds to the initial rate. Reactions were quenched by the addition of ammonium hydroxide to the final concentration of 0.1 M and the released MU was quantified by the fluorescence. Excitation and emission wavelengths were set to 360 nm and 450 nm, respectively. Calibration curves were made with known *Tr*Cel7A concentrations. *Tr*Cel7A used as a reference was treated identically with samples, but the BC was omitted. In the presence of BSA no binding of *Tr*Cel7A to reaction vessels and filters was observed.

### Measurement of the total bound *Tr*Cel7A

BC (0.01 – 1 g/L) was equilibrated with *Tr*Cel7A (10 nM – 15 µM) in 0.5 mL total volume for 30 min. The sample was transferred to 2 mL polypropylene syringe and pressed through a glass microfibre filter (Whatman GF-D) to separate cellulose free and bound *Tr*Cel7A [21]. The filtrate was centrifuged (2 min 10,000×g) to remove any solids that had passed through the filter and β-glucosidase (final concentration 4 nM) was added to 0.4 mL of the supernatant. After overnight incubation with β-glucosidase [*Tr*Cel7A]_Free_ was measured by MUL hydrolyzing activity. [*Tr*Cel7A]_Bound_ was found as a difference between the total concentration of *Tr*Cel7A and [*Tr*Cel7A]_Free_ and was expressed in nanomoles per gram BC.

### Measurement of the active site mediated binding of *Tr*Cel7A

Here the initial rates of MUL hydrolyzing activity of *Tr*Cel7A were measured in the presence of BC. BC (1 g/L) was incubated with *Tr*Cel7A (10 nM – 2.0 µM) and β-glucosidase (0.85 µM) in 0.5 mL total volume at 25°C. At selected time 5 µL of 0.5 mM MUL was added, incubated further (at 25°C) for defined time and quenched by the addition of ammonium hydroxide to the final concentration of 0.1 M. BC was separated by centrifugation (2 min 10,000×g) and the released MU was quantified by the fluorescence of the supernatant. Times of the MUL addition and incubation with MUL were selected depending on the concentration of *Tr*Cel7A but the total duration of the experiment was always 30 min. [*Tr*Cel7A]_FA_ was found from the rate of MUL hydrolysis using standard curves made without BC. [*Tr*Cel7A_Bound-OA_] was found as a difference between the total concentration of *Tr*Cel7A and [*Tr*Cel7A]_FA_. In the experiments with higher *Tr*Cel7A concentrations (1.0 µM – 15 µM) another model substrate, pNPL (0.5 mM final concentration), was used instead of MUL. Here the released para-nitrophenole was measured by the absorbance at 420 nm [21]. At 1.0 µM and 2.0 µM total *Tr*Cel7A concentrations [*Tr*Cel7A]_Bound-OA_ was measured in parallel experiments using MUL and pNPL model substrates. No differences between using MUL or pNPL were observed within the measurement error limits. In constructing the binding isotherm for *Tr*Cel7A_Bound-OA_ the [*Tr*Cel7A]_Free_ measured in the parallel experiments made under the same total *Tr*Cel7A and BC concentrations (see measurement of the total bound *Tr*Cel7A) were used instead of [*Tr*Cel7A]_FA_.

### Binding reversibility - dilution experiment

Binding reversibility of both *Tr*Cel7A_Bound_ and *Tr*Cel7A_Bound-OA_ after dilution was assessed.

For the reversibility on the level of *Tr*Cel7A_Bound_, 1 g/L BC was incubated with 1.0 µM *Tr*Cel7A and 2.5 µM β-glucosidase in 0.5 mL total volume for 30 min after what 4.5 mL buffer was added. At defined times after dilution 0.5 mL samples were withdrawn, BC was separated by filtration and [*Tr*Cel7A]_Bound_ was measured as described in “Measurement of total bound *Tr*Cel7A”.

For the reversibility on the level of *Tr*Cel7A_Bound-OA_, 1 g/L BC was incubated with 1.0 µM *Tr*Cel7A in the presence of 2.5 µM β-glucosidase in 0.5 mL total volume. After 30 min, 4.5 mL MUL in buffer was added (final concentration 10 µM). At defined times after dilution 0.5 mL samples were withdrawn and mixed with 0.5 mL of 0.2 M ammonium hydroxide to quench the MUL hydrolysis. BC was separated by centrifugation and [MU] was quantified by the fluorescence. [*Tr*Cel7A]_FA_ was calculated from the rate of MUL hydrolysis in the presence of BC using standard curves made without BC [31]. [*Tr*Cel7A_Bound-OA_] was found as a difference between total concentration of *Tr*Cel7A and [*Tr*Cel7A]_FA_.

### Binding reversibility - free enzyme depletion experiment

BC (0.1 g/L) was incubated with *Tr*Cel7A (30 – 100 nM) for 10 min in 0.5 mL total volume. BC was pelleted by centrifugation (2 min, 10,000×g), 0.45 mL of the supernatant was withdrawn and BC pellet was resuspended with 0.45 mL fresh buffer. 10 min after resuspension the cellulose bound and free *Tr*Cel7A were separated by filtration and [*Tr*Cel7A]_Bound_ was measured as described in “The measurement of total bound *Tr*Cel7A”.

### Measuring the release of *Tr*Cel7A from cellulose pellet

BC (0.1 g/L) was incubated with 30 nM *Tr*Cel7A in 0.5 mL total volume for 10 minutes. BC was pelleted by centrifugation (2 min 10,000 × g) and after defined time of standing with cellulose pellet 0.4 mL of supernatant was withdrawn and added to 5 µL of β-glucosidase (final concentration 4 nM). After incubation overnight with β-glucosidase the concentration of free *Tr*Cel7A in supernatant was measured by MUL hydrolyzing activity. For the zero time point the cellulose bound and free *Tr*Cel7A were separated by filtration through glass microfibre filter (Whatman GF-D) [21]. The filtrate was centrifuged (2 min 10,000×g) and after incubation with β-glucosidase [*Tr*Cel7A]_Free_ was measured by MUL-ase activity.

### Data treatment

The binding data were analyzed using non-linear regression analysis according to the following equations.

Langmuiŕs independent binding site(s) model
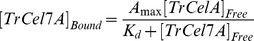



*A*_max_ is the binding capacity of BC (nmol/g BC) and *K*_d_ is the equilibrium dissociation constant of BC-*Tr*Cel7A complex (nM). In the case of Langmuiŕs two and three independent binding site models the hyperbolas containing the parameters for second (*A*_max(2)_, *K*_d(2)_) and third (*A*_max(3)_, *K*_d(3)_) binding mode were added to the right hand side of the equation.

Freundlich model
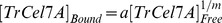
where *a* and 1/*m* are Freundlich equilibrium constant and power term, respectively.

Hilĺs model
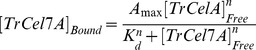
where *n* is cooperativity parameter. In all equations [*Tr*Cel7A]_Bound_ and [*Tr*Cel7A]_Free_ represent the concentration of cellulose bound (in nmol/g BC) and free (in nM) *Tr*Cel7A.

## Supporting Information

File S1 **This file contains following figures: Figure S1.** Release of *Tr*Cel7A from cellulose pellet after centrifugation. Figure S2. Binding kinetics at different concentrations of *Tr*Cel7A and BC. Figure S3. Binding of *Tr*Cel7A to BC and best fits of Langmuiŕs two and three binding sites model. Figure S4. Hill's plots for the binding of *Tr*Cel7A to BC in the high and medium affinity binding mode. Figure S5. *A*_max_ and *K*_d_ values for the binding of *Tr*Cel7A in the high affinity binding mode at different BC concentrations.
(PDF)
