# Supplementary material for: Multi-Mode Binding of Cellobiohydrolase Cel7A from Trichoderma reesei to Cellulose
Source: PLoS One. 2014 Sep 29;9(9):e108181. doi: 10.1371/journal.pone.0108181 (PMC4180464; doi:10.1371/journal.pone.0108181)
Supplement: File S1 — This file contains following figures: Figure S1. Release of TrCel7A from cellulose pellet after centrifugation. Figure S2. Binding kinetics at different concentrations of TrCel7A and BC. Figure S3. Binding of TrCel7A to BC and best fits of Langmuiŕs two and three binding sites model. Figure S4. Hill's plots for the binding of TrCel7A to BC in the high and medium affinity binding mode. Figure S5. A max and K d values for the binding of TrCel7A in the high affinity binding mode at different BC concentrations. (PDF) [file pone.0108181.s001.pdf]

Supplemental material to

Multi-mode binding of cellobiohydrolase Cel7A from *Trichoderma reesei* to cellulose

Jürgen Jalak and Priit Väljamäe*

Institute of Molecular and Cell Biology, University of Tartu, Riia 23b – 202, 51010 Tartu, Estonia.

* Corresponding author: e-mail: priit.valjamae@ut.ee

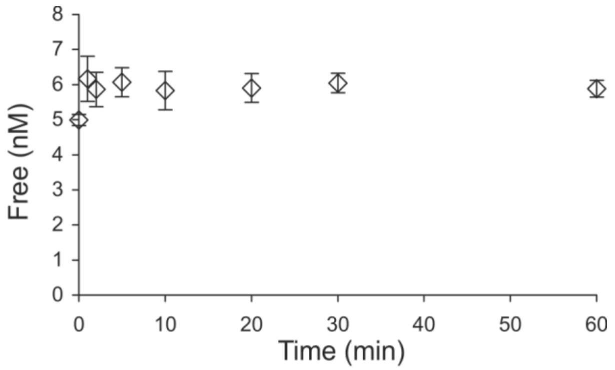

Figure S1. Release of *Tr*Cel7A from cellulose pellet after centrifugation. BC (0.1 g/L) was incubated with 30 nM *Tr*Cel7A for 10 min. BC with bound *Tr*Cel7A was pelleted by centrifugation (2 min at 10,000 *g*) and the concentration of free *Tr*Cel7A in supernatant was measured at different times after centrifugation. For zero time point the cellulose bound and free *Tr*Cel7A was separated by filtration.

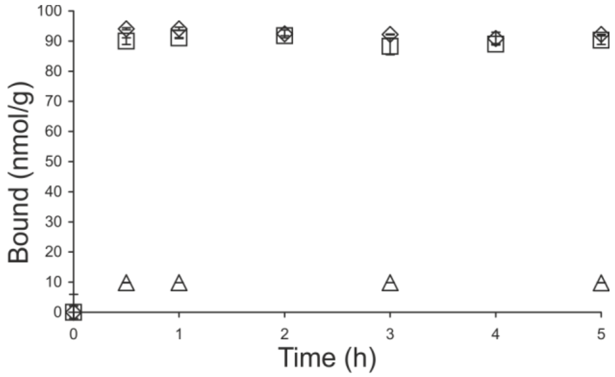

Figure S2. Binding kinetics at different concentrations of *Tr*Cel7A and BC. 0.1 μM *Tr*Cel7A and 1.0 g/L BC (◇), 0.01 μM *Tr*Cel7A and 0.1 g/L BC (☐), and 0.01 μM *Tr*Cel7A and 1.0 g/L BC (△).

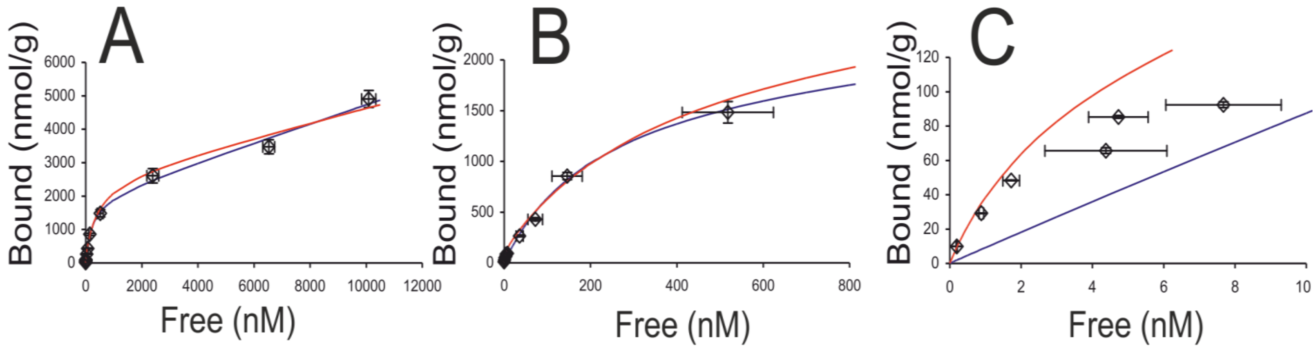

Figure S3. The presence of at least three binding sites must be assumed to describe the binding of *Tr*Cel7A to BC. Binding isotherm is dissected (as in Figure 1 of the main article) to show the regions with low (A), medium (B), and high (C) affinity dominating binding modes. Solid lines represent the best fit according to the Langmuir´s two (blue line) and three (red line) binding sites model. Although the two sites model is sufficient to describe the binding in the low (A) and medium (B) affinity region, it fails to account for the high affinity binding (C). The presence of at least three binding modes was further confirmed by distinguishing between different populations of bound enzyme (Figure 1 of main article).

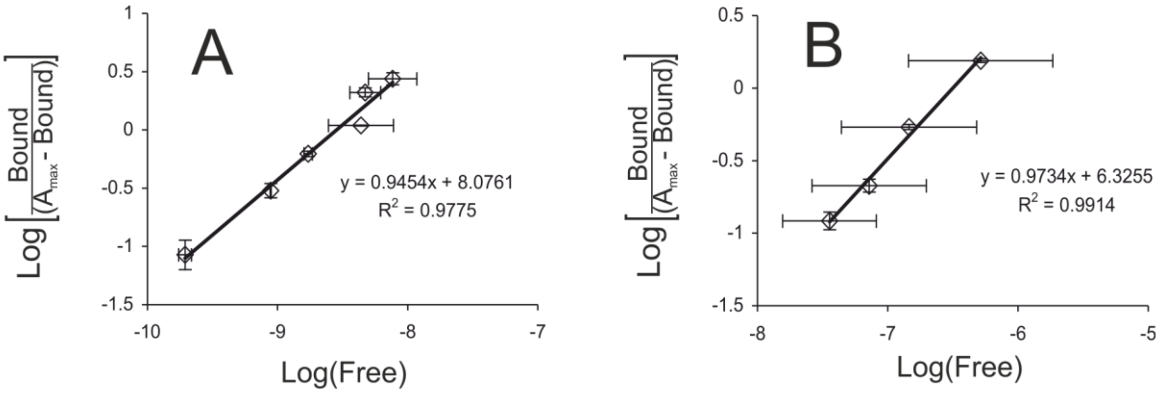

Figure S4. Hills plots for the binding of *Tr*Cel7A to BC in the high (A) and medium (B) affinity binding mode. The A_{max} values of 126 nmol/g and 2440 nmol/g were used to construct Hills plots for high and medium affinity binding mode, respectively. Solid lines represent the best fit according to the linear function. For both binding mode the slope of the line is close to 1 indicating no cooperativity within these binding modes.

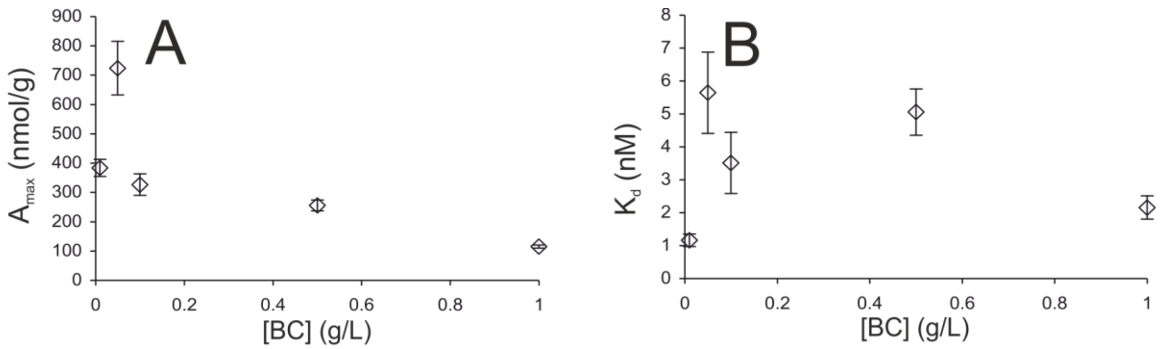

Figure S5. A_{max} and K_d values for the binding of *Tr*Cel7A in the high affinity binding mode at different BC concentrations. Binding data (original data are presented in panel A of Figure 4 in the main article) were analyzed according to the Langmuir´s one binding site model to get the A_{max} and K_d values.
